# Enzymatically Built Nanoenabled Antimicrobial Coating on Urinary Catheters

**DOI:** 10.1021/acsami.4c08599

**Published:** 2024-07-23

**Authors:** Antonio Puertas-Segura, Angela Gala Morena, Silvia Pérez Rafael, Kristina Ivanova, Ivan Ivanov, Katerina Todorova, Petar Dimitrov, Gianluca Ciardelli, Tzanko Tzanov

**Affiliations:** † Grup de Biotecnologia Molecular i Industrial, Department of Chemical Engineering, Universitat Politècnica de Catalunya, Rambla Sant Nebridi 22, 08222 Terrassa, Spain; ‡ Institute of Experimental Morphology, Pathology and Anthropology with Museum, Bulgarian Academy of Sciences, Geo Milev, 1113 Sofia, Bulgaria; § Department of Mechanical and Aerospace Engineering, Politecnico di Torino, Corso Duca degli Abruzzi 24, 10129 Torino, Italy

**Keywords:** silver-lignin nanoparticles, carboxybetaine, laccase, antimicrobial, antifouling, urinary catheters

## Abstract

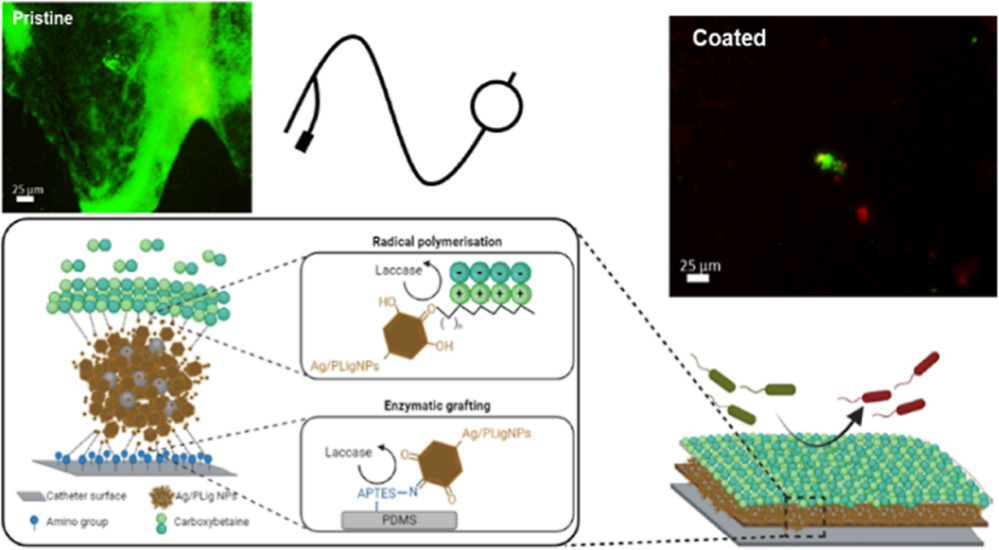

Catheter-associated urinary tract infections represent a major share of nosocomial infections, and are associated with longer periods of hospitalization and a huge financial burden. Currently, there are only a handful of commercial materials that reduce biofilm formation on urinary catheters, mostly relying on silver alloys. Therefore, we combined silver-phenolated lignin nanoparticles with poly(carboxybetaine) zwitterions to build a composite antibiotic-free coating with bactericidal and antifouling properties. Importantly, the versatile lignin chemistry enabled the formation of the coating in situ, enabling both the nanoparticle grafting and the radical polymerization by using only the oxidative activity of laccase. The resulting surface efficiently prevented nonspecific protein adsorption and reduced the bacterial viability on the catheter surface by more than 2 logs under hydrodynamic flow, without exhibiting any apparent signs of cytotoxicity. Moreover, the said functionality was maintained over a week both in vitro and in vivo, whereby the animal models showed excellent biocompatibility.

## 1 Introduction

Urinary catheters increase the risk of bacteriuria and potentially severe infection due to the development of biofilms, which, in parallel, can clog the device and compromise its proper function. Catheter-associated urinary tract infections (CAUTI), in fact, belong to the most-frequent hospital-acquired diseases.^1,2^ The prevention and management strategies include avoidance of catheterization or frequent replacement and antibiotic therapies,^3^ whose factors contribute to patient discomfort and emergence of drug-resistant bacteria. The latter complication escalates the treatment intensity and causes additional side effects,^4^ while aggravating the antimicrobial resistance on a global scale.

Nowadays, silicone catheters are standard and fairly safe in the short term, but the rapid nonspecific adsorption of biomolecules (forming the so-called conditioning layer) leads to inevitable bacterial colonization and therefore complicates the long-term use. Within the first 24 h of catheterization, bacteria usually form single-species biofilms, which in turn prompt the development of multispecies biofilms, altogether causing infections in 10–50% of the patients during ∼7 days of catheterization.^5,6^ An emerging approach to prevent biofilm formation is coating the catheters with antimicrobial and/or antifouling components such as metal nanoparticles (NPs),^7^ modified biopolymers,^8^ or zwitterions;^9,10^ but only few studies showcase comprehensive functional characterization, encompassing endurance tests and performance in more realistic environment.

In this context, silver nanoparticles (AgNPs) have been extensively studied as an alternative to traditional antibiotics for their broad antimicrobial properties.^11^ Though the traditional synthetic routes to produce AgNPs involve harsh chemical reagents that may contaminate the particles, which, coupled to the inherent toxicity of silver,^12^ has raised concerns about the possible safety in humans. On the other side, lignin is a largely available antibacterial and antioxidant biopolymer from biomass^13,14^ that can be used both to reduce silver ions and stabilize the NPs.^15,16^ Thus, the nanoformulation of metals with lignin yields a phenolic shell around the particles, which not only promotes their stability but also creates a reactive surface that enables multiple interactions in the composite materials. Metal/lignin particles present broad-range antibacterial activity and, thanks to the multifaceted and unspecific antimicrobial mechanisms, hinder the appearance of drug resistance.^17,18^ In this regard, we have previously synthesized lignin-capped AgNPs with pronounced activity against several pathogenic strains^19^ and demonstrated that lignin reduced the inherent toxicity of silver, while simultaneously enhancing the effects against bacteria.^18,20,21^

Here, we sought to engage these improved functionalities in a coating for urinary catheters in order to ensure effective and prolonged shielding against biofilms. Importantly, we augmented the antibacterial activity of silver-phenolated lignin nanoparticles (AgPLigNPs) with the antifouling properties of zwitterions, intervening at the initial stage of biofilm formation. To this end, we covalently immobilized both actives onto the catheter material via laccase oxidation. This eco-friendly approach circumvents some of the disadvantages of chemical grafting and polymerization,^22−24^for example, by being more selective, controllable, and carried out under mild reaction conditions, with water as the sole byproduct.^25,26^ The oxidized phenolic substrates yield highly reactive phenoxy radicals that can either reorganize into quinone structures, form covalent bonds with other molecules, or spontaneously polymerize.^10,27^ In line with this multiple reactivity, in the present work laccase simultaneously grafted AgPLigNPs onto an aminated silicone surface and initiated the polymerization of carboxybetaine methacrylate (CBMA) monomers. The resulting coatings were thoroughly characterized by several spectroscopic and imaging methods, while the silver loading and release kinetics were assessed via inductively coupled plasma mass spectrometry (ICP-MS). In parallel, their activity was measured via antimicrobial and antibiofilm tests against *Pseudomonas aeruginosa* and *Staphylococcus aureus*,^28^ next to unspecific protein adsorption. Finally, the biocompatibility and realistic performance were evaluated through in vitro measurements of cytotoxicity and a week-long implantation in rabbits.

## 2 Experimental Section

### 2.1 Materials

Urinary catheters and material specimens of polydimethyl/vinylmethyl siloxane (also known as VMQ; further abbreviated in text as PDMS) from Degania product line were supplied by QMD Catheter Technologies. Protobind 6000 lignin (∼90% sulfur-free, average molecular weight ∼1000 g/mol, obtained from annual plants) was purchased from Green Value (Switzerland). 3′,5′-Dimethoxy-4′-hydroxyacetophenone (acetosyringone), tannic acid, and gallic acid (GA) were obtained from ACROS Organics. Ethanol was purchased from Scharlab. Sodium acetate, acetic acid, petroleum ether, phosphate-buffered saline (PBS), Muller Hinton (MHB), Baird-Parker agar, cetrimide agar, Dulbecco’s modified Eagle’s medium, sodium dodecyl sulfate (SDS), 2,2-dihydroxyindane-1,3-dione (ninhydrin), fluorescein isothiocyanate-labeled bovine serum albumin (FITC-BSA), and crystal violet were purchased from Sigma-Aldrich. *N*,*N*-Dimethylaminoethyl methacrylate (DAEM) and β-propiolactone were purchased from ThermoFisher. Live/Dead BacLight kit (molecular probes L7012) and alamarBlue cell viability reagent were obtained from Invitrogen. Fungal laccase Novozym 51003 from *Myceliophthora thermophila* (EC 1.10.3.2) was supplied by Novozymes. Its activity was 13.2 U/mL, where 1 U converted 1 μmol/min ABTS in 0.05 M sodium acetate buffer (pH 5 at 25 °C). Ultrapure water from Millipore was used in all experiments. Biofilm-proficient bacteria cultures, *S. aureus* (ATCC 25923) and *P. aeruginosa* (ATCC 10145) were aerobically grown at 37 °C in a tryptic soy broth (TSB).

### 2.2 Preparation of CBMA Monomers

CBMA monomer was synthesized in one step by the reaction of DAEM and β-propiolactone, according a previously described protocol.^29^ First, a 50% (v/v) solution of DAEM in 2-butanone was generated and cooled at 5 °C. Then, a solution of β-propiolactone 0.25 g/mL in 2-butanone at a 6:1 ratio was gradually added for 3 h at 5 °C in an ice bath. The solution was kept for 12 h at 5 °C to complete the synthesis. The finished monomers were then cleaned with diethyl ether and allowed to dry for 24 h at 25 °C. The obtained product was characterized using attenuated total reflectance-Fourier transformed infrared spectroscopy (FTIR-ATR) (PerkinElmer/FTIR Spectrum 100R) and ^13^C NMR (Bruker NMR Ascend 400 MHz).

### 2.3 Preparation and Characterization of AgPLigNPs

AgPLigNPs were synthesized as previously described.^19^ An aqueous solution of the phenolated lignin (10 mg/mL) at pH 8 was mixed with 4 mg/mL AgNO_3_ at a 3:2 lignin/silver volume ratio and ultrasonicated for 2 h at 60 °C (VCX 750 Ti-horn, 20 kHz, 50% amplitude). The obtained AgPLigNPs were centrifuged at 20,000*g* for 20 min, then resuspended in water and centrifuged at 500*g* for 10 min to remove the unreacted lignin, and finally the NPs were dispersed by low-intensity ultrasound. The surface charge of the particles was assessed by a Zetasizer Nano Z (Malvern Instruments Inc.). Transmission electron microscopy (TEM) was performed on a JEOL JEM-2100 LaB6 instrument operating at 200 kV and coupled with energy dispersive X-ray (EDX) spectroscopy. Size distribution was extracted using ImageJ. The antimicrobial properties of AgPLigNPs were assessed through MIC by using resazurin-based assay.^30^ The presence of phenols was assessed through a previously described spectrophotometric assay.^19^

### 2.4 PDMS Functionalization

Pieces (1 × 1.5 cm) of PDMS in laminar form (the actual material used for production of urinary catheters) were washed in 0.1% (w/v) SDS solution, water, and ethanol. The PDMS pieces were treated with plasma using an O_2_ at 13.56 MHz and 100 W for 10 min. After the treatment, the samples were introduced into a solution of 5% APTES (v/v) in ethanol at room temperature for 24 h and then washed with ethanol. The presence of amino groups on the PDMS surface was confirmed by a ninhydrin test using 2% (w/v) solution, which caused a distinctive color shift. Then PDMS samples treated with APTES were incubated in acetate buffer (pH 5, 0.05 M) containing 1.5 mg/mL of acetosyringone, 13.12 U/mL of laccase, and 30% (v/v) of AgPLig-NPs in a laboratory shaker at 50 °C for 1.5 h at 350 rpm. Subsequently, the carboxybetaine monomers (1 M) were added and the final solution was incubated at 50 °C for 22.5 h at 350 rpm. Finally, samples were washed with water to eliminate the unbound reagents and materials.

### 2.5 Characterization of PDMS Coatings

#### 2.5.1 Attenuated Total Reflectance-Fourier Transformed Infrared Spectroscopy

ATR-FTIR spectra of different silicone samples were recorded on a Spectrum 100 FTIR spectrometer (PerkinElmer) at a 4 cm^–1^ resolution.

#### 2.5.2 X-Ray Photoelectron Spectroscopy

Spectra were recorded with a pass energy of 25 eV at pressure < 6 × 10^–9^ mbar using XR50 source and Phoibos 150 MCD-9 detector, while further experimental details have been reported before^31^ C 1s spectra at 285 eV were used for reference. The surface composition was determined by using the sensitivity factors provided by the manufacturer.

#### 2.5.3 Scanning Electron Microscopy

The surface of the samples was observed by scanning electron microscopy (SEM) using a field-emission scanning electron microscope at 1 kV (Merlin Zeiss). The elemental composition of the material surface was analyzed by EDX.

#### 2.5.4 Atomic Force Microscopy

The surface morphology of pristine and coated samples was assessed on a Dimension 3100 instrument (Veeco) in tapping mode. The data was analyzed using Nanotec WSxM.^32^

#### 2.5.5 Water Contact Angle

The wettability was measured by the sessile drop method on a DSA 25 (Krüss) using Krüss Advanced v1.13.0.21301 software and the tangential method to calculate the water contact angle (WCA).

#### 2.5.6 Determination of Silver Content and Release Profile

The silver release was followed by Model 7800 ICP-MS system from Agilent by immersing 1 × 1 cm PDMS samples in 2 mL of artificial urine (pH 6.8, UNE EN1616)^31^ incubated at 37 °C and 100 rpm and exchanged every 24 h. The solution samples were stored at 4 °C; then 1 mL of 2% HNO_3_ was added prior to analysis.

#### 2.5.7 Stability of the Coating

To determine the durability of the coatings and their functionality upon longer indwelling, contact angle, and antibiofilm tests were carried out for freshly coated samples and samples incubated in artificial urine under mild agitation (100 rpm) at 37 °C for a week.

### 2.6 Antimicrobial and Antifouling Analysis

#### 2.6.1 Protein Adsorption Tests

Samples from different coating stages were immersed in 1 mg/mL of FITC-BSA in water for 30 min. This mimicked the immediate attachment of a protein before biofilm formation. Then the samples were rinsed and dried with nitrogen. The attached protein was detected on a NIKON/Eclipse Ti–S fluorescence microscope.

#### 2.6.2 Antimicrobial Properties

The antimicrobial activity was evaluated following the method ASTM-E2149-01. Single *S. aureus* and *P. aeruginosa* colonies were grown in 5 mL of MHB at 230 rpm and 37 °C overnight and then diluted with PBS to absorbance at 600 nm equal to 0.28. Then, the solution was diluted thousand-fold in PBS and each PDMS sample was incubated with the bacterial suspension (1.5 mL). To determine the inoculum cell density, the suspensions were withdrawn after 24 h of incubation with samples, and the surviving bacteria were counted by plating on cetrimide and Baird–Parker agar depending on the strain.

#### 2.6.3 Antibiofilm Properties

To determine the live bacterial cells inside the biofilm in static conditions, *S. aureus* and *P. aeruginosa* were grown in sterile TSB for 24 h at 37 °C. Each sample was inoculated in a 24-well sterile plate with 1 mL of suspended bacteria (0.01 OD in TSB) and the samples were incubated for 24 h at 37 °C. After washing three times with PBS to remove the nonattached bacteria, samples were transferred into 2 mL of PBS in 15 mL tubes. Then, the tubes were vortexed for 2 min, sonicated for 20 min, and viable counts were assessed after plating. In parallel, a qualitative assessment of biofilm formation on the material surface was conducted using the Live/Dead BacLight kit. In this regard, fluorescence micrographs were captured at 480_ex_/500_em_ nm for Syto 9 and 490_ex_/635_em_ nm for propidium iodide.

#### 2.6.4 Hydrodynamic Model of Catheterized Bladder

An in vitro model of catheterized human bladder was used to assess the ability of the coating to inhibit the development of biofilm under dynamic settings.^33^ Briefly, Foley catheters were inserted into the model and fixed by inflation of the balloon with 5 mL of PBS. The bladder was filled with sterile artificial urine, and 1 mg/mL *P. aeruginosa* and *S. aureus* in TSB (OD_600_ = 0.01) were added. The model was maintained for a week at 37 °C at a perfusion rate of 1 mL/min. Upon catheter removal, bacterial viability was assessed by colony counting.^31^

### 2.7 Biocompatibility of the Coatings

Human foreskin fibroblasts (BJ-5ta, ATCC-CRL4001) were maintained according to the supplier instructions.^31^ Prior to biocompatibility evaluation, cells were seeded at 1.2 × 10^5^ cells/well on a tissue culture-treated polystyrene plate. Then silicone samples were cut into round pieces of 1 cm and placed in contact with the cells, and the reported protocol was followed.^31^ Toxicity was determined by AlamarBlue assay (Invitrogen), while the Live/Dead assay for mammalian cells revealed the morphology.

### 2.8 In Vivo Evaluation of Catheter Performance

New Zealand male rabbits aged 4–5 months (3–4 kg) were kept in individual cages with free access to food and water. All experimental procedures were carried out in accordance with the national regulation on laboratory animals and animal welfare (no. 20/01.11.2012), the 2010/63/EU directive of the European Parliament, and approved by the Ethical Committee of the Institute of Experimental Morphology, Pathology and Anthropology with the Museum (no. 282/24.09.2020). After 2 weeks of quarantine, the animals were examined and catheterized with pristine catheters (French size 8) (control group 1, *n* = 3) and treated catheters of the same size (experimental group 2, *n* = 3) for 7 days. Catheterization was done under general anesthesia, following the reported protocol.^31^ During surgery rabbits were disinfected and cervical collars were placed for protection. The animals were examined daily, and all recovered after the catheterization. Blood from the jugular vein and urine through catheters were collected before and after the experiment, while urine from the bladder was also taken after the euthanasia. Urine was preceded for urinalysis and microbiological tests as reported before, next to blood counts and biochemistry.^31^

After 1-week catheterization, all six rabbits were humanely euthanized and histological materials were taken from the urethra, bladder, and kidneys. The samples were fixed in 10% formalin, dehydrated, cleared in xylene, and embedded in paraffin. Tissue sections (3–5-μm thick) were stained with hematoxylin/eosin and examined with a Leica DM 5000B microscope for lesions and signs of inflammation or infection.

### 2.9 Statistical Analysis

All reported values are provided with their respective mean and standard deviation. Multiple comparisons were conducted using Graph Pad Prism Software version 5.04, employing a one-way analysis of variance followed by either a posthoc Tukey’s test or the unpaired two-tailed Student’s *t* test technique. Statistical significance was defined as *p*-values less than 0.05 (*), 0.01 (*), and 0.001 (***), representing varying degrees of significance in the statistical analysis.

## 3 Results and Discussion

### 3.1 Coating Precursors

The zwitterionic carboxybetaine monomer chemical structure was validated by FTIR-ATR (1167 cm^–1^ methacryloyl group, 1629 cm^–1^ carbon double bond, 1589 cm^–1^ stretching of carboxylate groups, 1718 cm^–1^ typical of ester groups and a large shoulder at 3300 cm^–1^ for hydroxyl groups,^34^Figure S1) and ^13^C NMR (600 MHz, D_2_O 298 K: 176.0, 168.5, 135.2, 127.8, 62.4, 62.0, 58.5, 51.4, 30.4, 30.3, and 17.3 ppm, Figure S2). TEM images of the AgPLigNPs sample revealed dispersed silver particles with an average size of 13 ± 3 nm, in a matrix corresponding to phenolated lignin (Figure S3a–c), while the ζ-potential was around −35 mV. EDX analysis confirmed the presence of Ag (Figure S3d). Phenolation and nanoformulation have been reported to enhance the antimicrobial properties of lignin^35^ but in this work potent antimicrobial effect was observed only with silver (Figure S4). A phenolic content assay quantified 162 ± 6 mg GA equivalents per g of NP sample, which residual phenols were used to initiate enzymatic grafting onto the aminated substrate and radical polymerization of the zwitterions.

### 3.2 Physicochemical Characterization of the Coating

**Figure 1:**
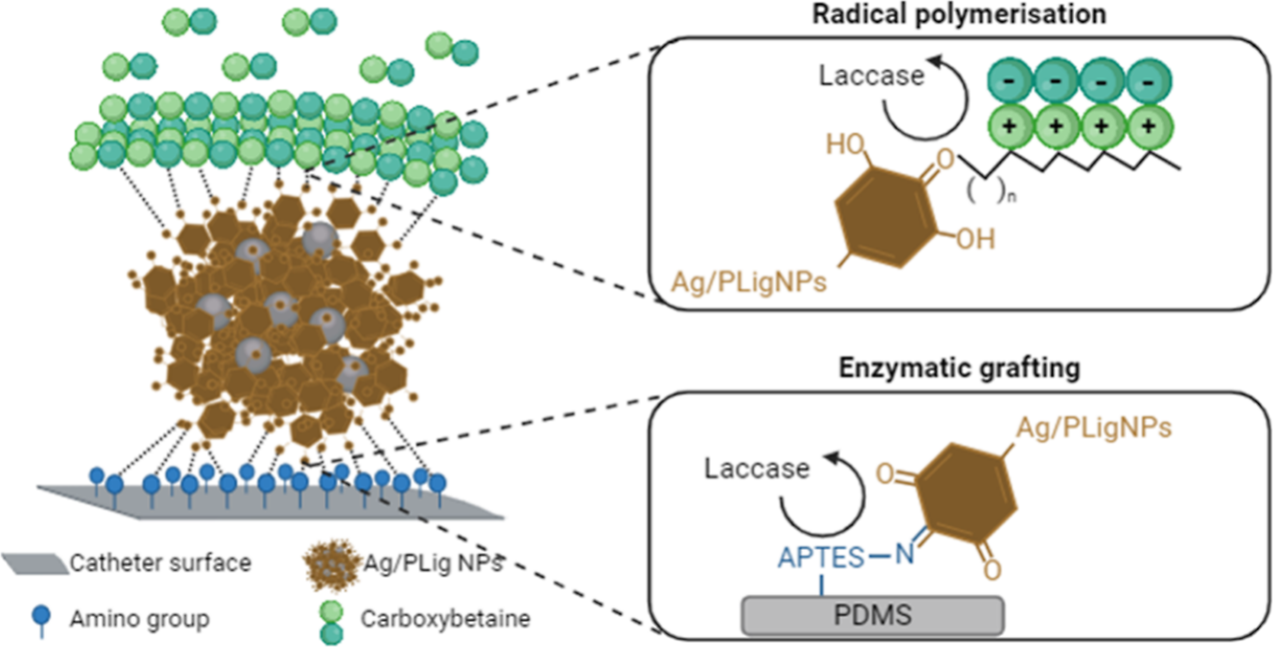
Scheme of the enzymatic approach for nanoenabled coating.

The formation of the coating comprised three steps: (i) amination of the silicone surface, (ii) enzymatic grafting of AgPLigNPs, and (iii) enzymatic polymerization of CBMA (Figure 1).

**Figure 2:**
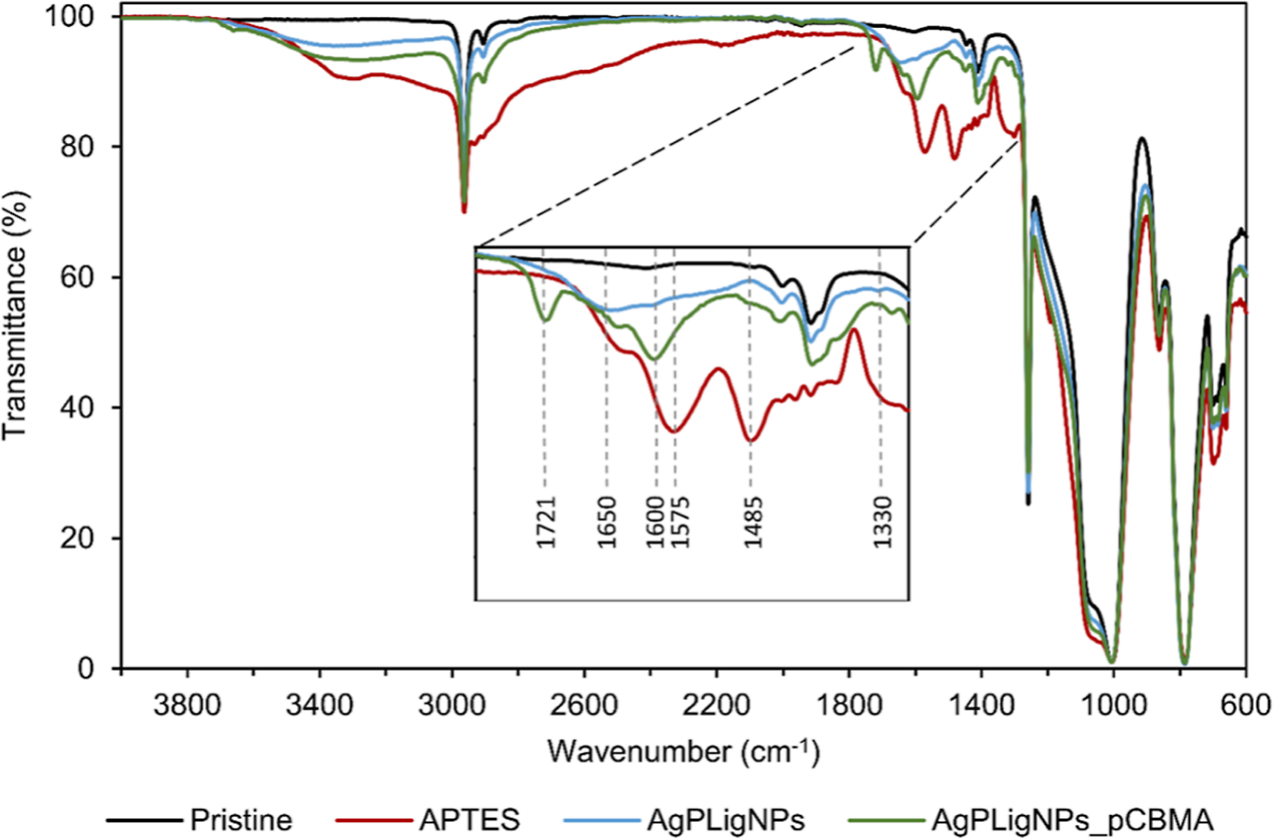
FTIR-ATR spectra recorded at each coating formation step: pristine PDMS (Pristine), aminated PDMS (APTES), AgPLigNPs-grafted PDMS (AgPLigNPs), and PDMS sample with a complete coating (AgPLigNPs_pCBMA).

After activation with oxygen plasma, chemisorption of APTES was used to generate anchoring points on the inert silicone surface.^36^ The peaks at 1485 and 1575 cm^–1^ (characteristic vibrational signals of amino species) in the FTIR spectra (Figure 2), together with the appearance of a new signal at 400 eV in the respective N 1s X-ray photoelectron (XPS) spectra, confirmed the presence of free nonprotonated amino groups (Figure S5 and Table S1). Additionally, the ninhydrin assay yielded the characteristic purple color upon reaction with the aminated surface (Figure S6).

The subsequent covalent grafting of AgPLigNPs on the surface was done by laccase. Enzymatic oxidation of the phenolic groups in the lignin shells produces highly reactive quinones, which react with nucleophilic amino groups. The successful modification with NPs led to the disappearance of the peaks at 1485 and 1575 cm^–1^ assigned to primary amino groups, while a new shoulder was detected between 1550 and 1650 cm^–1^ due to stretching of C=N bonds and the aromatic ring of quinones. The presence of the imine groups was related to a Michael addition and Schiff base covalent bond formation between *o*-quinones and amino groups from the coated surface.^10,25,37^ Additionally, the analysis of C 1s XPS spectrum after grafting AgPLigNPs revealed the formation of new C–H/C–H (285 eV) and C–O–C/C–OH (287 eV) from the different functional groups in the AgPLigNPs (Figure S7 and Table S2).

The final step of the coating formation consisted in the radical polymerization of carboxybetaine monomers, initiated by the same enzymatically generated phenoxy groups that had not intervened in the grafting process of the AgPLligNPs. The corresponding FTIR spectra confirmed the presence of CBMA by the appearance of new peaks at 1600 and 1721 cm^–1^ due to carboxylate and C=O stretching, respectively. This was further confirmed by the presence of a new peak in the XPS spectra assigned to the quaternary ammonium in CBMA (Figure S5 and Table S1).

**Figure 3:**
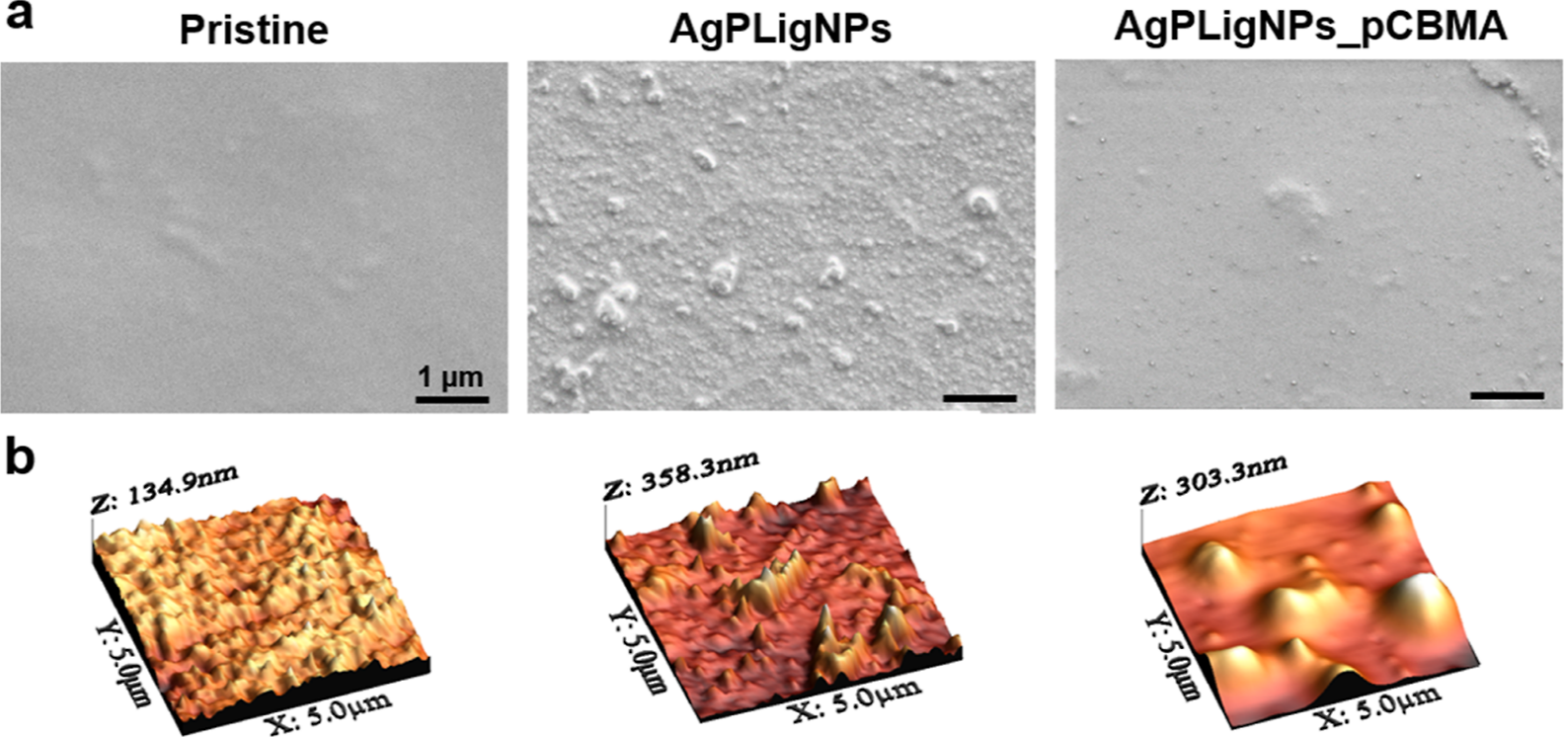
(a) SEM micrographs of pristine silicone, grafted with AgPLigNPs, and coated with polyzwitterions (AgPLigNPs_pCBMA). (b) Representative 3D morphologies of the silicone surfaces at each coating formation step.

The surface morphology following the different steps of the coating formation was investigated using SEM and atomic force microscopy (AFM) (Figure 3), except for the sample after amination due to the molecular dimensions of the APTES layer. The surface heterogeneity increased considerably after grafting of AgPLigNPs (both root-mean-square roughness *R*_Q_ and average *R*_A_ values from AFM image analysis increased more than 2-fold), corroborating a successful immobilization.

Therefore, larger aggregates were ascribed to the high reactivity of the oxidative phenol derivatives, causing additional cross-linking of the lignin matrices. Interestingly, carboxybetaine polymerization apparently smoothened the surface, giving rise to a continuous top layer, although this was not reflected in the *R*_A_ and *R*_Q_ values, which remained around 40–50 nm. Thus, despite being discrete nodes, the AgPLigNPs enabled a homogeneous zwitterion coating. Additionally, the presence of AgPLigNPs and carboxybetaine was confirmed by EDX (Figure S8). The potential influence of surface roughness on bacterial adhesion is not fully established and there are conflicting reports,^38^ likely due to the complex interplay with other surface properties. Interestingly, systematic increase of *R*_A_ might lead to varying bacterial coverage as recently reported, revealing a sweet spot for attachment at intermediate values.^39^ On the other side, lubricity unequivocally decreases with increasing roughness, yet the formal data from force–distance measurements has not been related to patient comfort, while the present *R*_A_ values of the AgPLigNPs_pCBMA coating lie well within the reported ones for commercial catheters.^40^

### 3.3 Functional Characterization

#### 3.3.1 Protein Adsorption

**Figure 4:**
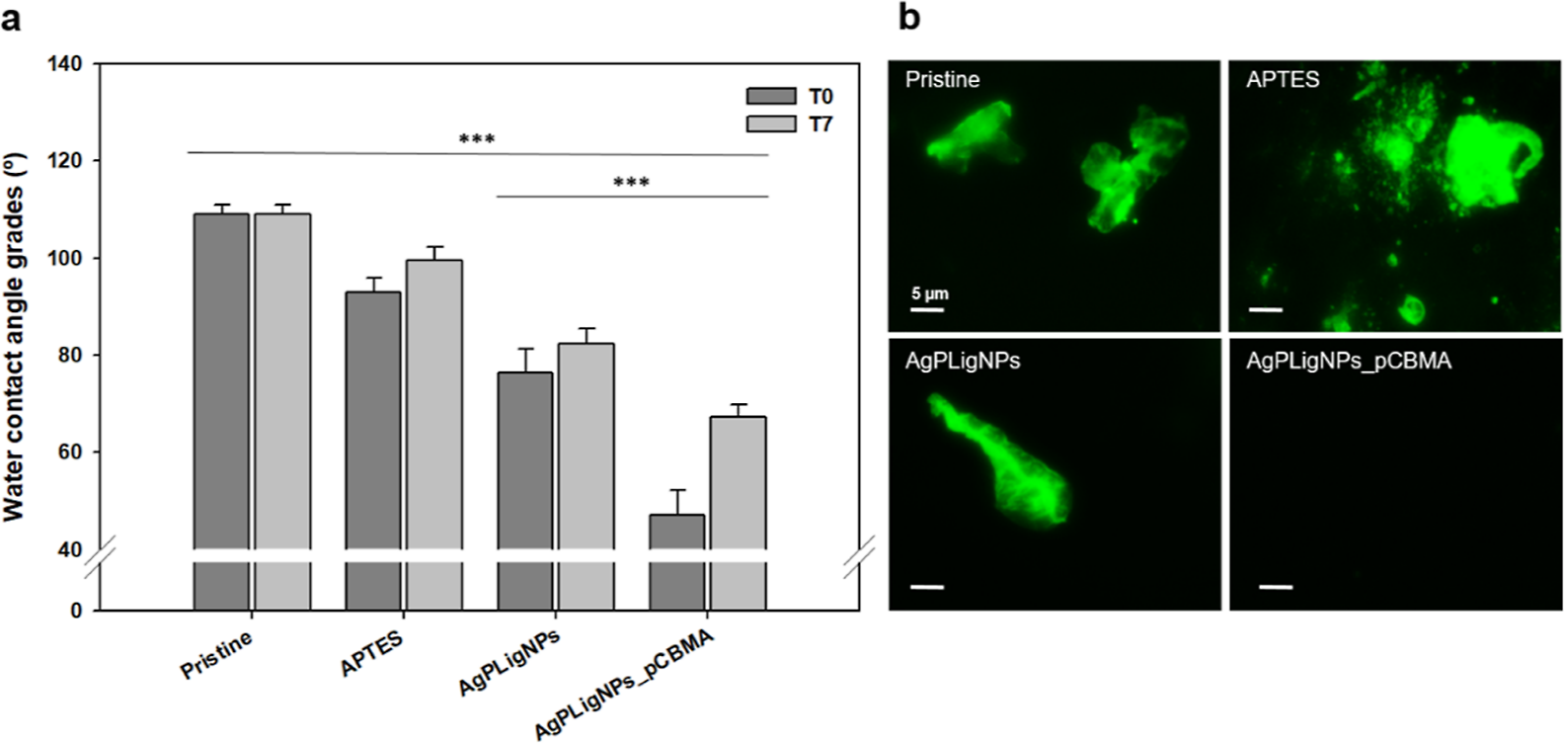
(a) WCA measurements before and after 7 days of incubation at each step of coating formation (*n* = 10). (b) Fluorescence images taken after 30 min of incubation in 1 mg/mL of FITC-labeled BSA solution (*n* = 3).

A recurring symptom in patients with urinary tract infections is the presence of plasma proteins in their urine. These proteins nonspecifically adhere to hydrophobic surfaces such as silicone, facilitating bacterial attachment and subsequent biofilm development.^41^ Increased hydrophilicity of the catheter surface results in a close connection with the surrounding water, preventing hydrophobic proteins from contacting the material.^42^ The hydrophilic properties of the coatings and their durability were therefore assessed by contact angle measurements after 1 week of incubation in synthetic urine at 37 °C (Figure 4a). The wetting gradually improved upon each functionalization step. After grafting AgPLigNPs, the contact angle decreased by about 30°, which was ascribed to the increased roughness, and the hydrophilic phenol and hydroxyl groups on the surface.^43,44^ The polycarboxybetaine layer resulted in further increase in hydrophilicity, in line with the reported values for this material.^45,46^ Overall, the contact angle values did not change considerably upon incubation, and even though the zwitterionic coating was least stable, it maintained high wettability after a week.

Next, the silicone samples were incubated with labeled BSA in the same solution to simulate the protein adhesion during catheterization. Notably, conditioning layer was formed on all surfaces apart from the one with a polyzwitterionic coating (Figure 4b). This confirmed that mere hydrophilicity was not sufficient to ward off the unspecific adsorption of biomolecules and corroborated the superior antifouling functionality of zwitterions thanks to the establishment of a hydration layer through electrostatic interactions.^46^

#### 3.3.2 Antimicrobial Activity against Planktonic Bacteria

**Figure 5:**
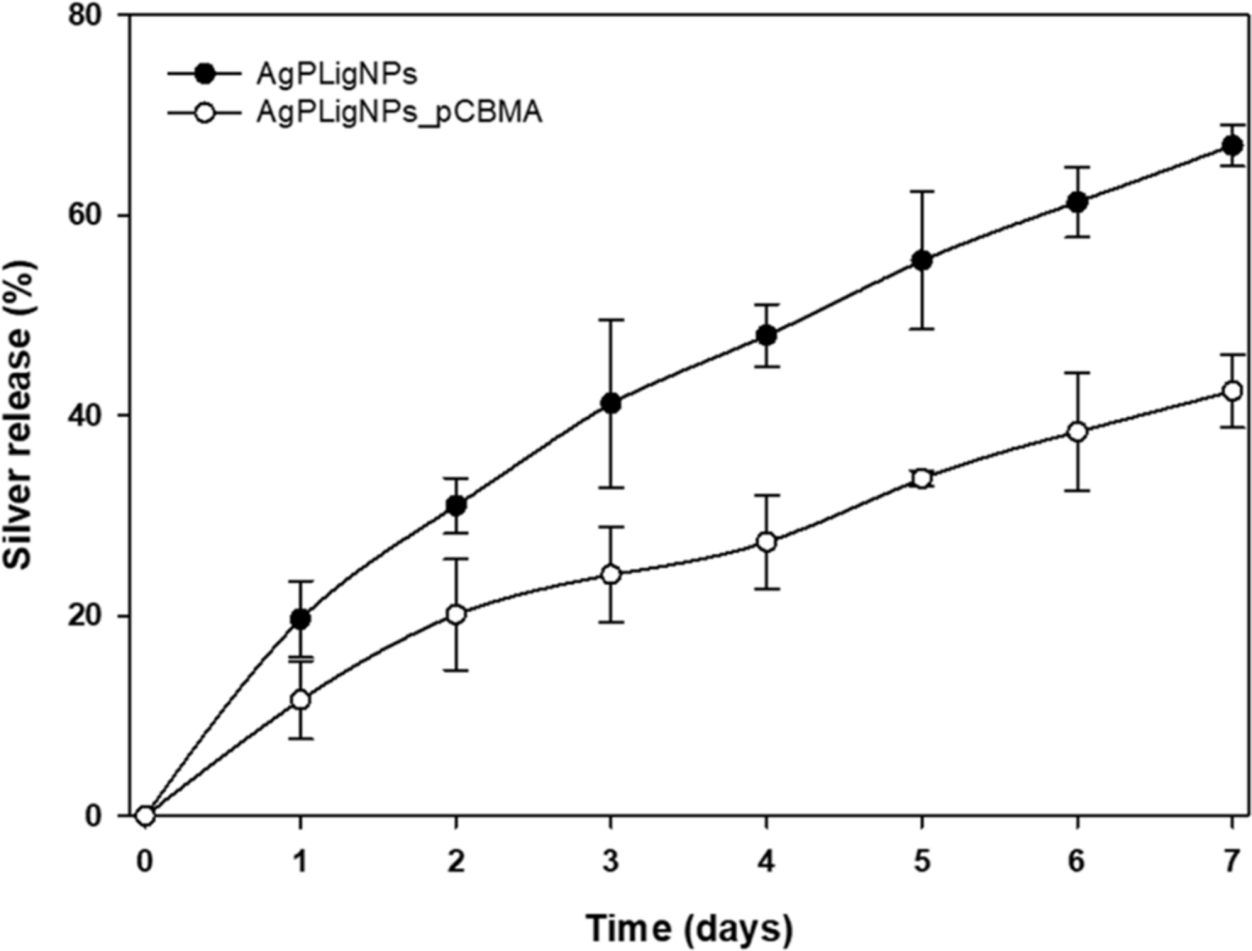
Silver release from 1 × 1 cm of coated PDMS samples after incubation in 2 mL of artificial urine over 7 days at 37 °C (*n* = 3).

Since the main antimicrobial agent in the nanoenabled coating was silver, its amount and release rate were deemed critical for the efficacy and the durability. On 1 × 1 cm samples coated with AgPLigNPs and AgPLigNPs_pCBMA, the silver loading was around 34 and 46 μg, respectively, as determined by ICP-MS. This agreed with the experimental protocol, which allowed for additional NP trapping during the polymerization. In both cases, no burst release was observed and the kinetics appeared as first order (Figure 5). Thus, the zwitterionic layer, in addition to providing an antifouling effect, enhanced the loading of silver during coating formation. Moreover, pCBMA slowed the release by forming a dual barrier together with the lignin matrix, maintaining 60% of residual silver loading after a week-long incubation.

**Figure 6:**
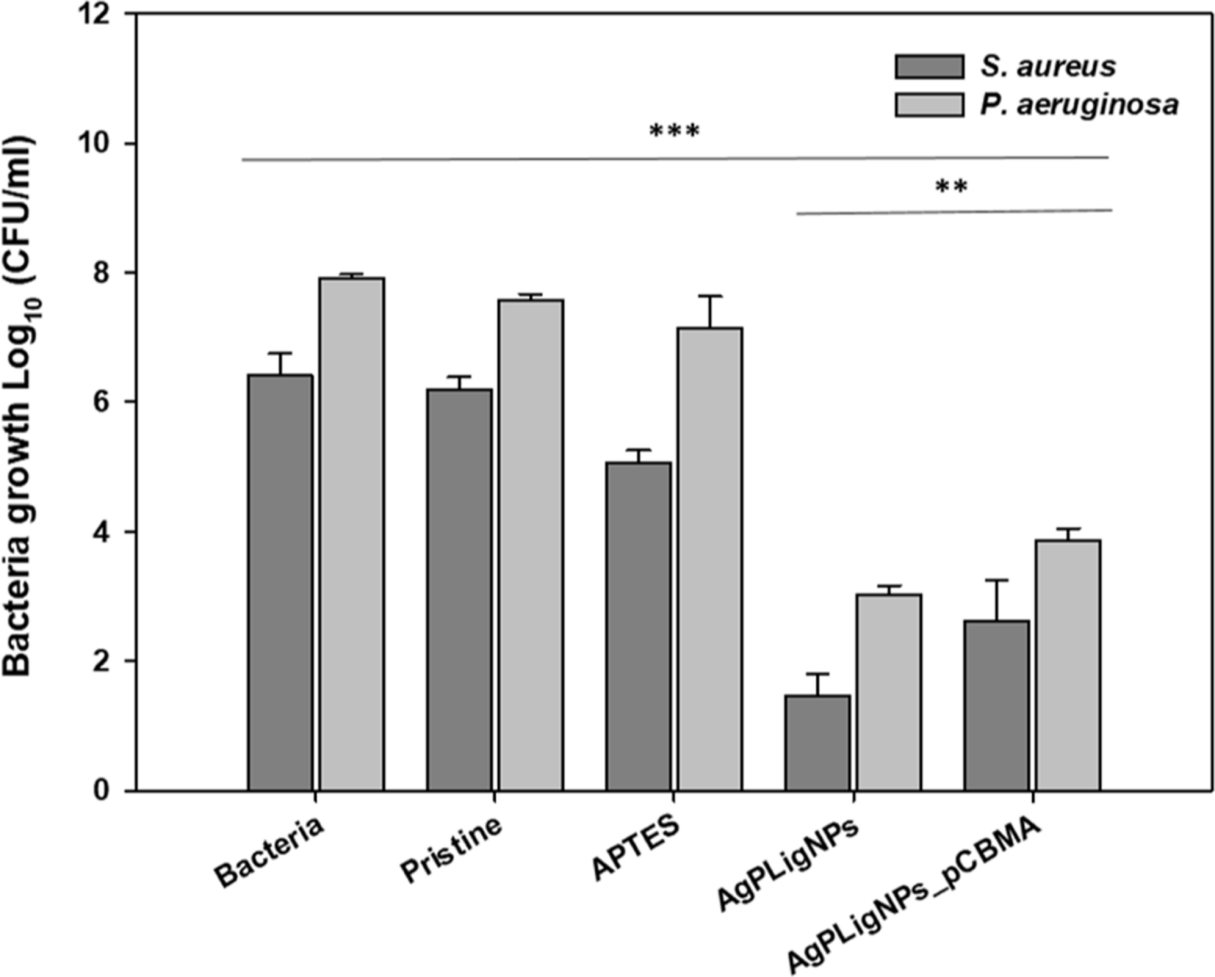
Antibacterial activity of the coated silicone materials against *P. aeruginosa* and *S. aureus* after 24 h of incubation at 37 °C (*n* = 3).

The antibacterial properties were then assessed against *P. aeruginosa* and *S. aureus*, prevalent in urinary tract infections (Figure 6). Grafting of AgPLigNPs alone resulted in more than a 4-log reduction of both pathogens after 24 h of incubation, surpassing the performance of the hybrid coating by about 1 log, which activity agreed well with the higher silver release in the absence of a polyzwitterionic layer.

#### 3.3.3 Antibiofilm Properties

**Figure 7:**
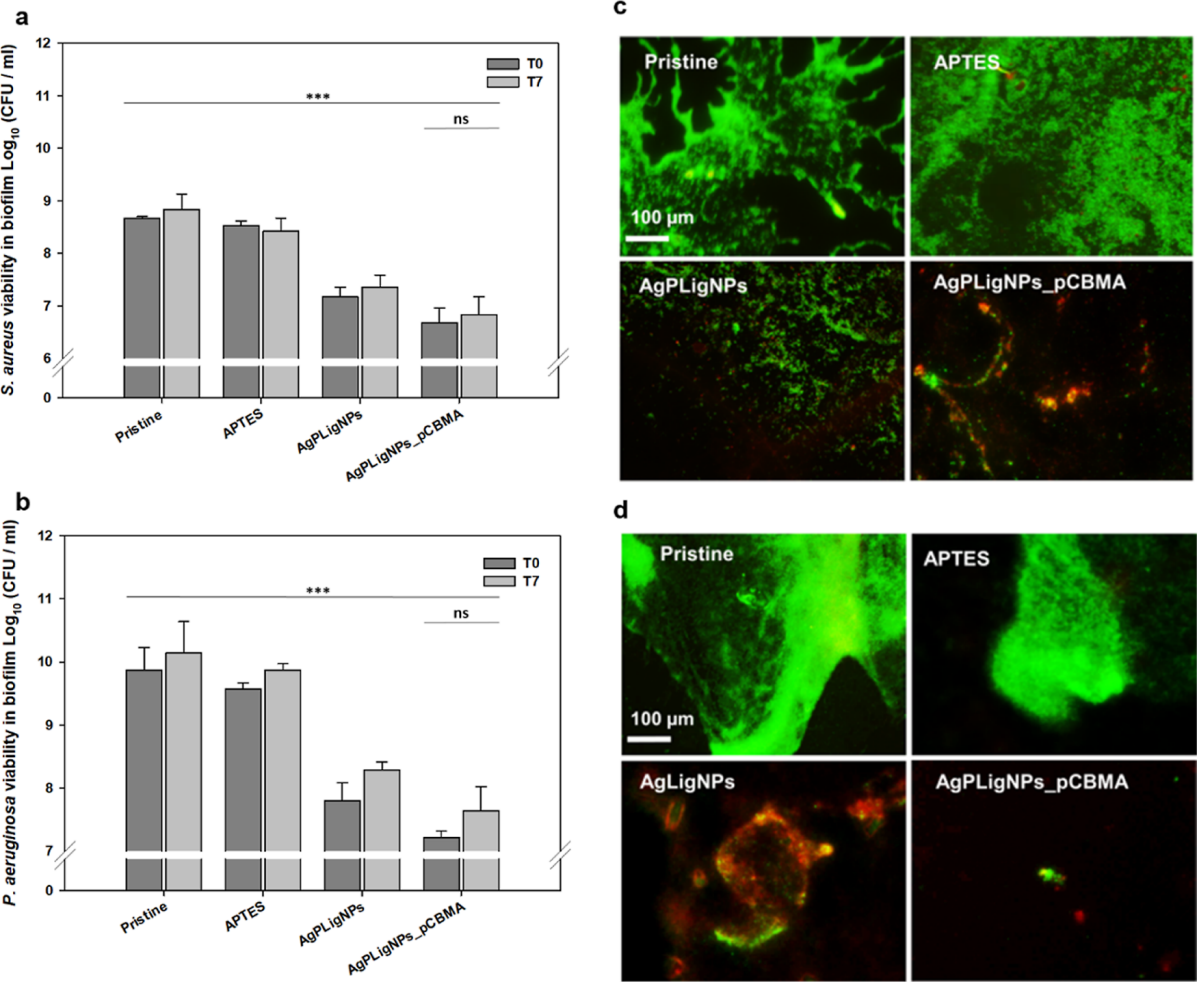
Cell viability of *S. aureus* (a) and *P. aeruginosa* (b) in biofilms on untreated and coated silicones at static conditions after 7 days of incubation in artificial urine (*n* = 3); fluorescence microscopy images of live (green) and dead (red) *S. aureus* (c) and *P. aeruginosa* (d) in biofilms on pristine and coated PDMS.

The prevention of *P. aeruginosa* and *S. aureus* biofilm formation was assessed by incubation with bacterial cultures at static conditions using bacterial viability assay,^33^ mimicking the initial conditions for biofilm formation after catheterization. The combined effect of the antimicrobial AgPLigNPs and the antifouling polycarboxybetaine led to 2-log reduction, which activity was mirrored after 7 days (Figure 7a,b), confirming the durability of the coating on the PDMS surface.

Furthermore, biofilm formation and bacterial viability were visually examined with a live/dead assay. Pristine and aminated surfaces exhibited a substantial amount of biofilm with a high viability. Coating with AgPLigNPs alone led to a noticeable reduction in viability attributable to the antimicrobial properties of silver; yet, the dead cells (and matrix) remained as debris on the surface. On the contrary, the establishment of a zwitterionic layer led to a nearly complete biofilm inhibition (Figure 7c,d).

**Figure 8:**
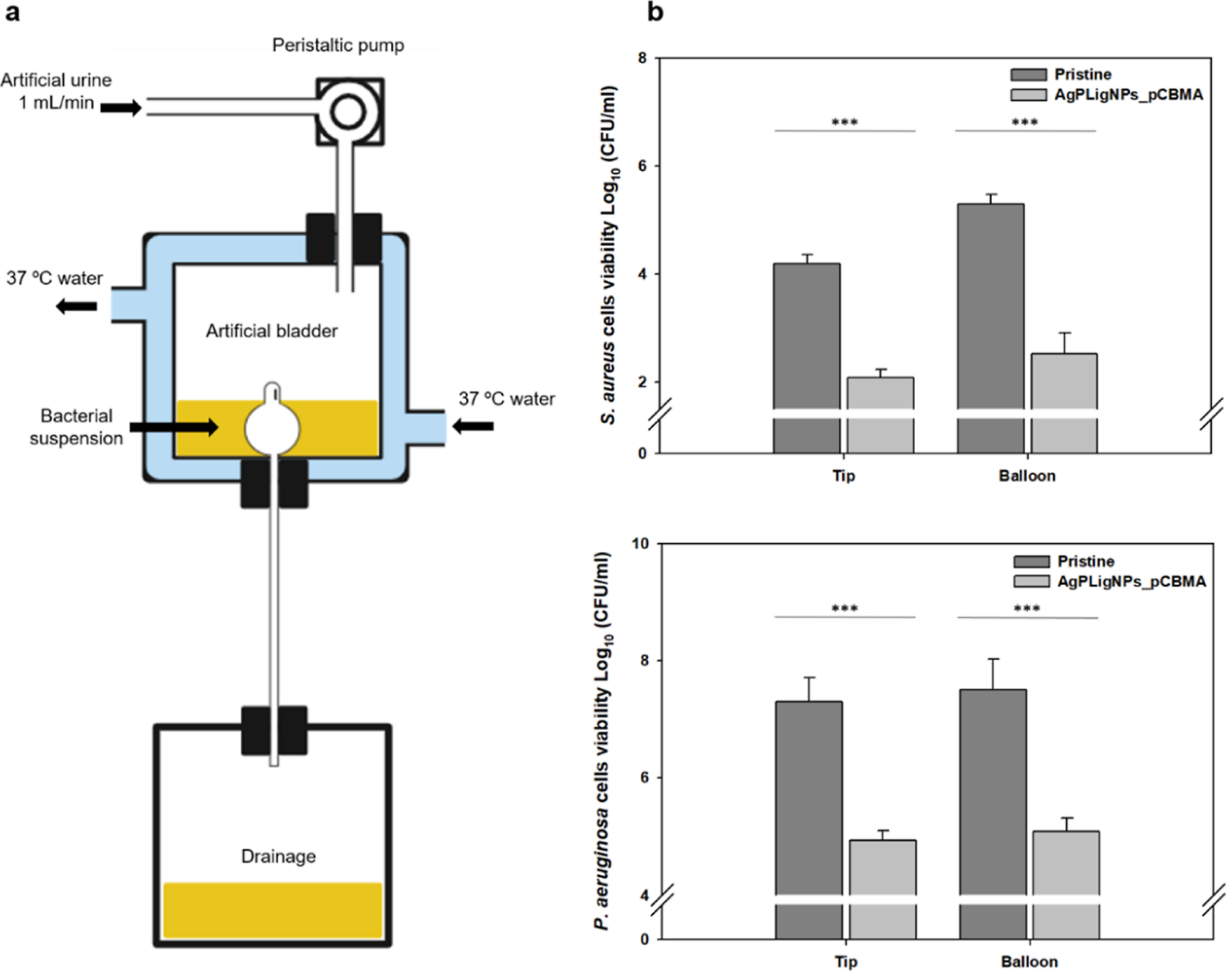
(a) Scheme of the dynamic assay procedure. (b) Amount of bacteria on urinary catheters after exposure to mixed bacterial culture during 7 days at 37 °C with perfusion (*n* = 3).

Finally, the durability was tested via an in-house dynamic setup (Figure 8a) designed to replicate the hydrodynamic conditions of the urinary tract. To this end, catheters were inserted into the artificial bladders, inoculated, and synthetic urine was perfused. The flow rate of 1 mL/min emulated unrestricted intraluminal flow in the presence of a drainage bag, and corresponded to the daily urine production of an adult (0.8–2 L).

Following a 7-day exposure period, the catheters were extracted, sectioned, and subjected to the previously outlined procedure to quantify bacterial adherence to the catheter surface. The investigation encompassed both individual (Figure S9) and combined (Figure 8b) bacterial cultures, the latter setup simulating more authentically the mixed-species burden encountered in infectious processes. A noteworthy reduction was evident in the treated catheters observed in both mixed and isolated cultures.

### 3.4 Cytotoxicity Evaluation

**Figure 9:**
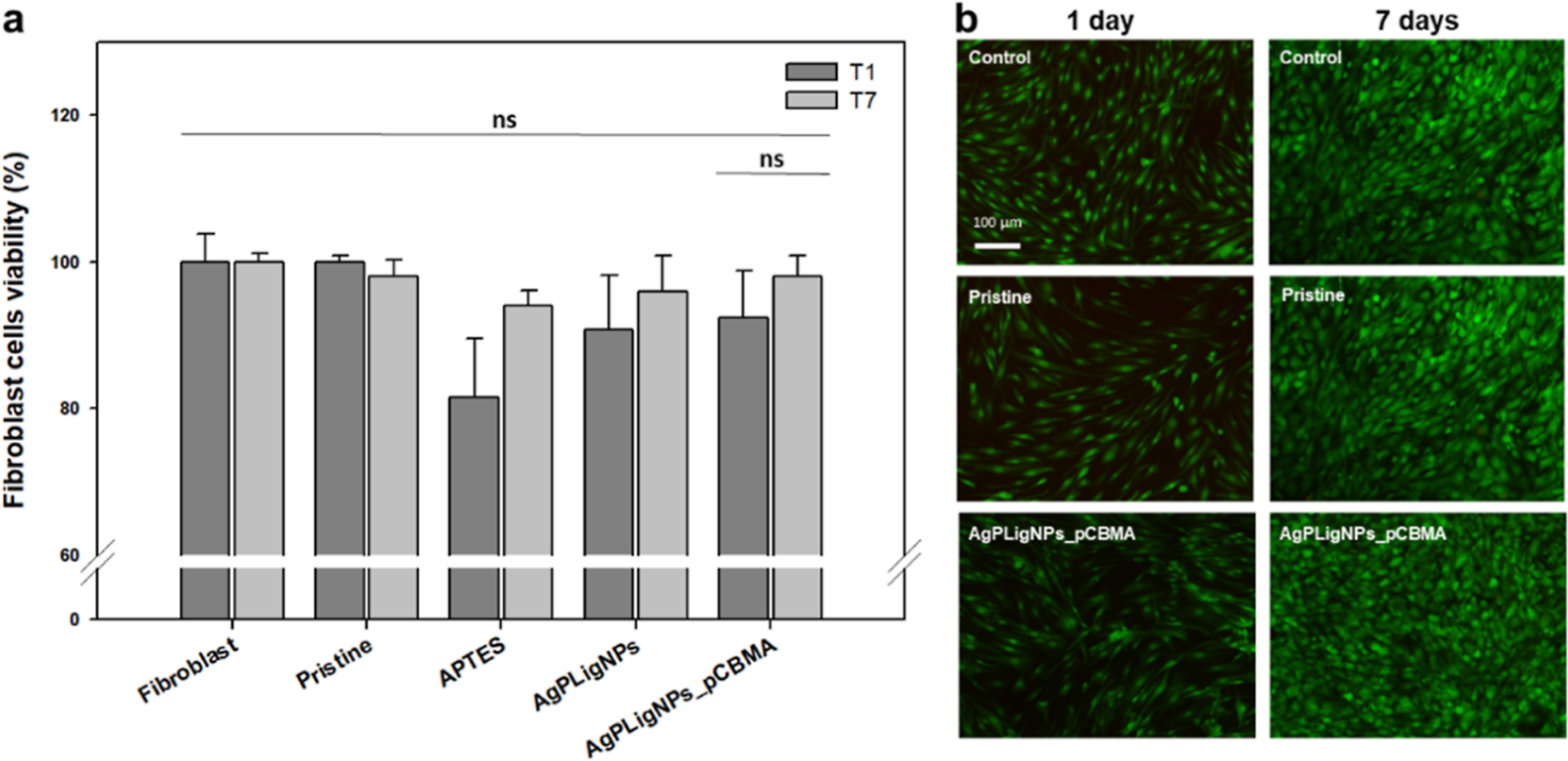
Viability of the BJ-5ta cell line after exposure to differently coated silicones assessed by (a) alamarBlue and (b) live/dead assays of AgPLigNPs_pCBMA-coated samples after different contact times. The green and red fluorescent images are overlaid (*n* = 3).

Biocompatibility is an essential parameter for the biomedical application of coatings. Silver has high antimicrobial efficacy due to its nonspecific activity; however, this broad-spectrum activity may also affect mammalian cells. In this regard, fibroblasts showed more than 90% viability after one and 7 days in direct contact with coated samples. The fluorescence images did not reveal any morphological changes either (Figure 9a,b).

### 3.5 In Vivo Assessment in a Rabbit Model

Following the encouraging results on antimicrobial performance and cytotoxicity, the hybrid coatings underwent final validation against untreated catheters in vivo. During the 7 -day catheter indwelling, the rabbits recovered and remained healthy, as hematological analysis of fundamental cell parameters indicated (Tables S3 and S4). On the day of catheterization, the values fell within the reference range. However, by the conclusion of the experiment (Table S4), diminished values for red blood cells and hemoglobin were recorded in both groups. Moreover, reactive thrombocytosis was noted in some instances. Blood biochemistry corroborated overall good health during catheterization (Table S5), with characteristic patterns of stress and discomfort, seen in comparable experimental designs.^31^ Some rabbits exhibited low creatinine and elevated creatine kinase due to minimized motility, while high sugar indicated stress. In parallel, malnutrition from stress and preventive usage of the collar might have led to lower urea nitrogen and amylase in all animals.

**Figure 10:**
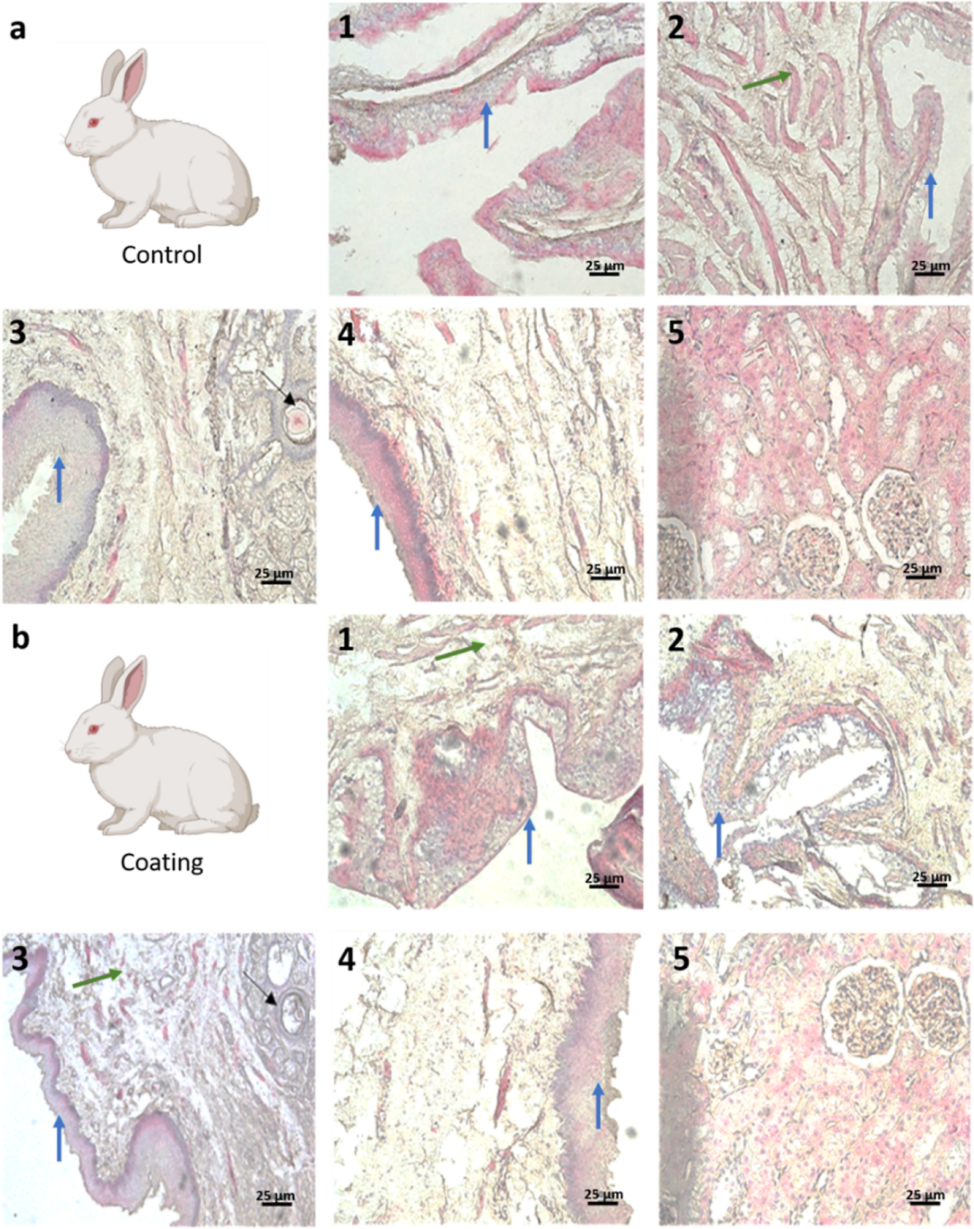
Microphotographs of the penile to the preprostatic part of a representative rabbit from the coated (a) and uncoated (b) group. 1–4: urethral parts: transitional epithelium (urothelium) (blue arrows) and surrounding layers of loose connective tissue, rich in elastin fibers and muscle tissue layers (green arrows), and black arrows point corpora amylacea in the prostatic parts of the urethra, 5: kidney parenchyma with renal corpuscles and convoluted tubules.

Microbiological assessment of urine directly collected from the bladder secured that the animals did not have bacteriuria at the beginning of the experiment. Upon its end, coated catheters displayed only minimal levels of *Enterococcus faecalis*, while pristine catheters exhibited significant quantities of both *E. faecalis* and *S. aureus*, pathogens frequently implicated in CAUTI. In addition, the elevated urine concentration of white blood cells in the control group suggested an immune response to an infection, likely induced by catheter colonisation,^47^ as well as mild excess of urobilinogen and bilirubin, hinting at potential liver issues. In parallel, after 7 days of catheterization, the histology of both groups did not show any morphological abnormality (Figure 10). Well-developed urothelium and intact fibromuscular stroma were present along the course of the urethrae. Renal cortex and medulla revealed normal renal corpuscles, convoluted tubules and collecting ducts. No histopathological lesions, erosions, or inflammatory cells were evident neither in urethra nor in the kidneys of any animals.

The thorough analysis of microbiological, clinical, and histological data gathered from the rabbit models substantiated the biocompatibility of the coated catheters and their effective prevention of CAUTI within 1 week of insertion. On the contrary, uncoated controls displayed bacterial colonization, which had likely induced an infection as evidenced also by elevated leukocyte esterase values. Importantly, the simultaneous load of *E. faecalis* and *S. aureus* in the control group emphasizes the rapid onset of colonization during catheterization, which poses a notable risk for severe inflammation, even if the histology did not apparently change.

## 4 Conclusions

Despite the long history and commercial availability of silver-based coatings on urinary catheters, the clinical trials are still inconclusive^48^ and there is no universal consensus or a guideline for their use. In this regard, hybridization of silver NPs with phenolated lignin leads to superior antimicrobial activity next to biocompatibility, yet the problem is how to form a robust functional coating in the challenging context of the urinary tract. Therefore, we augmented silver with the proven antifouling properties of zwitterions, in order to prevent bacterial colonization at the root cause level, prior to biofilm formation. Thereby, the available phenol chemistry allowed us to design a coating process that relies solely on an enzyme, both for the grafting of NPs on the surface and for the in situ polymerization of zwitterion precursors. The resulting nanoenabled coatings endowed the silicone surface with increased hydrophilicity, reduced protein adsorption, and sustained silver release within a clinically relevant experimental duration. Moreover, the coated catheters exhibited antimicrobial activity under hydrodynamic conditions mimicking human anatomy. Finally, animal studies corroborated the efficacy and reinforced the translational value of these nanocomposite materials in the context of CAUTI prevention.

## Supporting Information Available

FTIR-ATR spectra of carboxybetaine, NMR characterization of carboxybetaine, TEM/EDX images of NPs, bacteriostatic activity of NPs, XPS of coatings, SEM/EDX images of coatings, dynamic antibiofilm test, blood parameters (PDF)
