# Supplementary material for: Enzymatically Built Nanoenabled Antimicrobial Coating on Urinary Catheters
Source: ACS Appl Mater Interfaces. 2024 Jul 23;16(30):39129–39. doi: 10.1021/acsami.4c08599 (PMC11299133; doi:10.1021/acsami.4c08599)
Supplement: Supplementary file 1 — am4c08599_si_001.pdf [file am4c08599_si_001.pdf]

Supporting Information

Enzymatically built nanoenabled antimicrobial coating on urinary catheters

Antonio Puertas-Segura,[1] Angela Gala Morena,[1] Silvia Pérez Rafael,[1] Kristina Ivanova,[1] Ivan Ivanov,[1] Katerina Todorova,[2] Petar Dimitrov,[2] Gianluca Ciardelli,[3] Tzanko Tzanov*[1]

[1]*Grup de Biotecnologia Molecular i Industrial, Department of Chemical Engineering, Universitat Politècnica de Catalunya, Rambla Sant Nebridi 22, 08222, Terrasa, Spain*

[2]*Institute of Experimental Morphology, Pathology and Anthropology with Museum, Bulgarian Academy of Sciences, Geo Milev, 1113 Sofia, Bulgaria*

3Department of Mechanical and Aerospace Engineering, Politecnico di Torino, Corso Duca degli Abruzzi 24, 10129 Torino, Italy

*Corresponding author: tzanko.tzanov@upc.edu

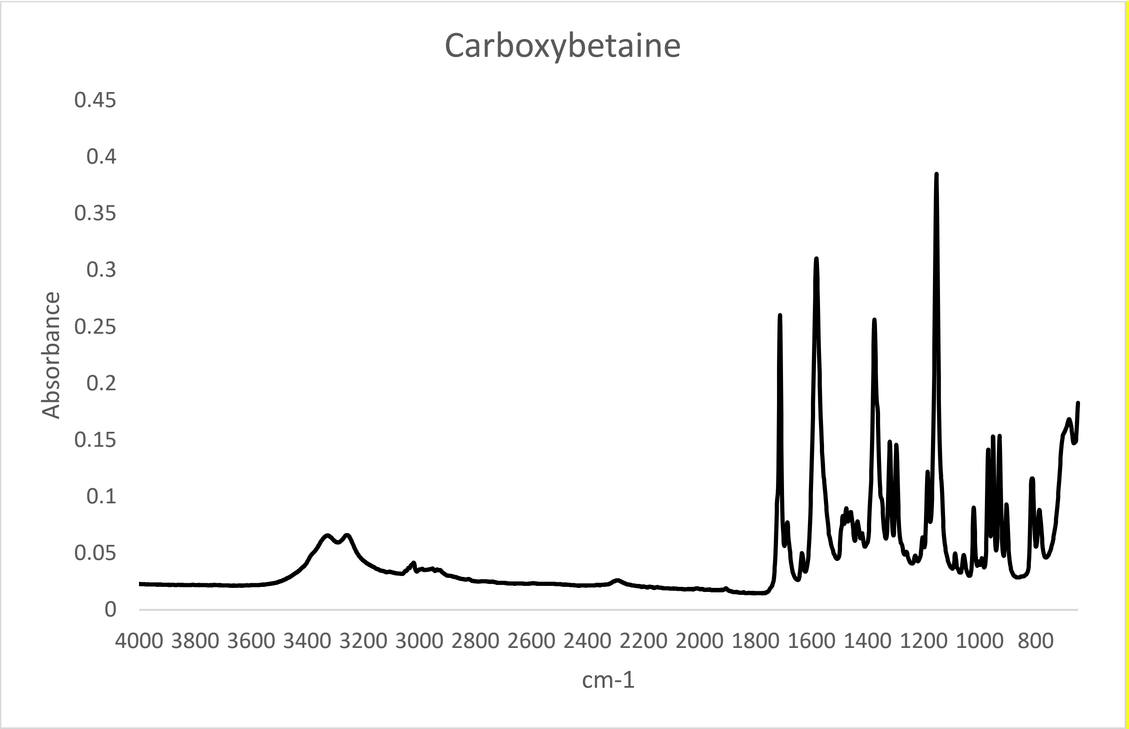

Figure S1. FTIR-ATR spectrum of a carboxybetaine methacrylate monomer.

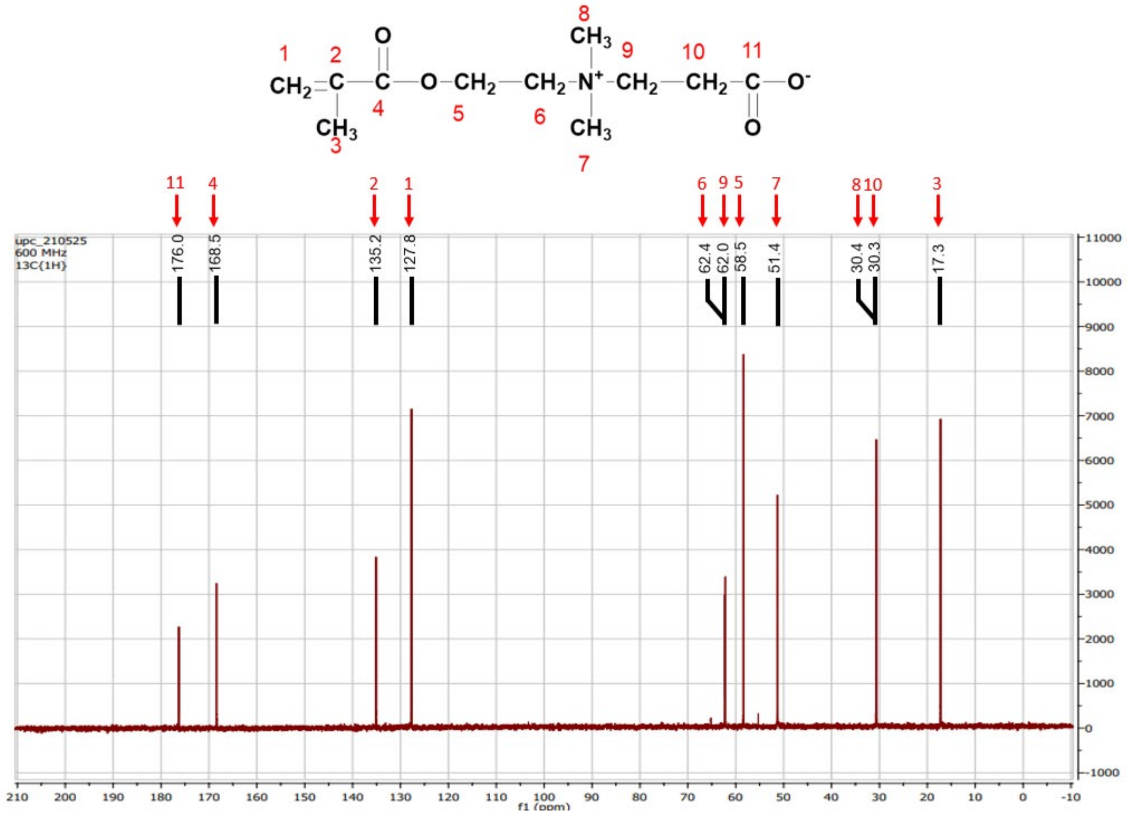

Figure S2. ^{13}C NMR spectrum of a carboxybetaine methacrylate monomer.

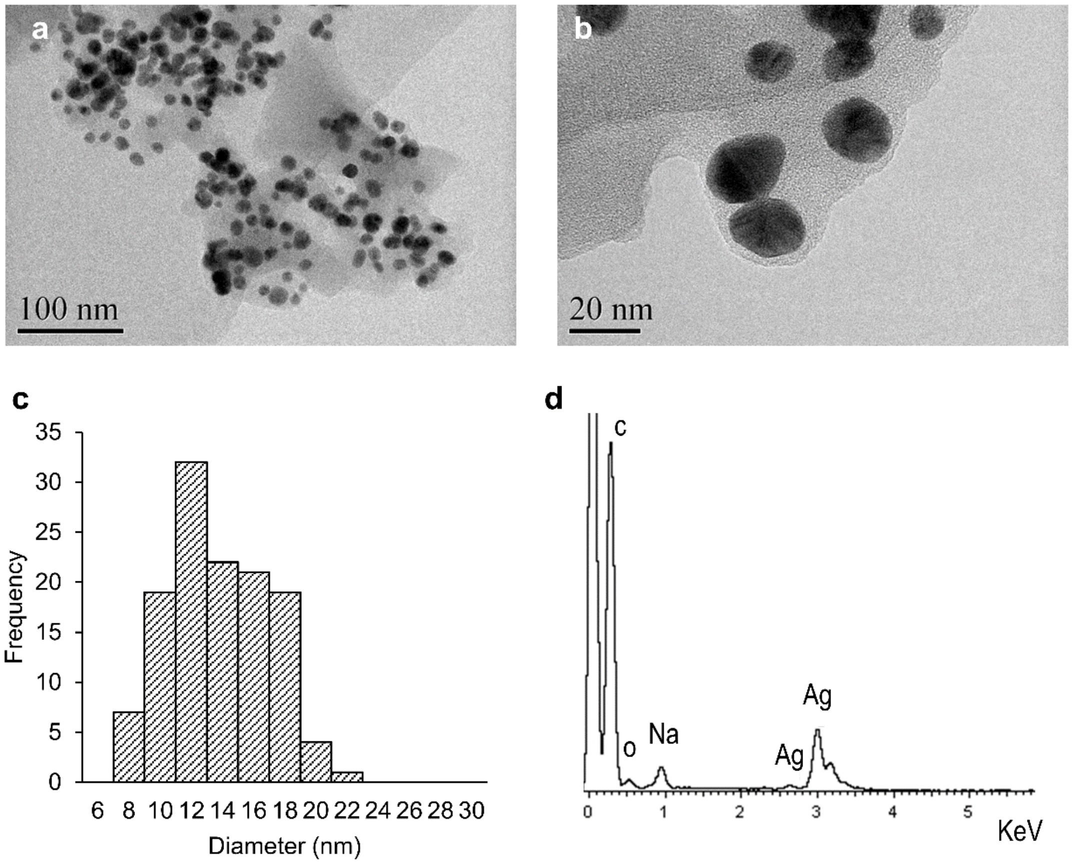

Figure S3. (a,b) TEM images of AgPLigNPs. c) Size average of AgPLigNPs from TEM data, d) EDX analysis of AgPLigNPs.

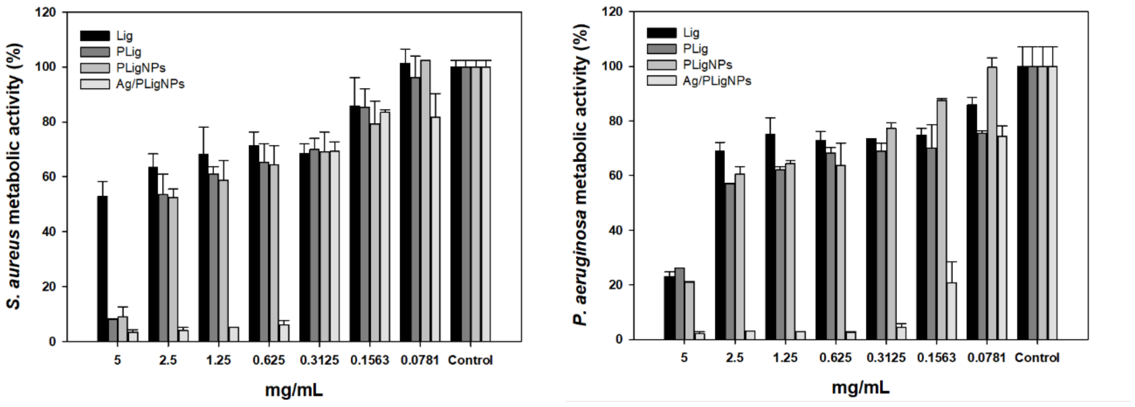

Figure S4. Antibacterial activity of lignin (Lig), phenolated-lignin (PLig), phenolated-lignin nanoparticles (PLigNPs) and silver phenolated-lignin nanoparticles (AgPLigNPs) against *S. aureus* and *P. aeruginosa*.

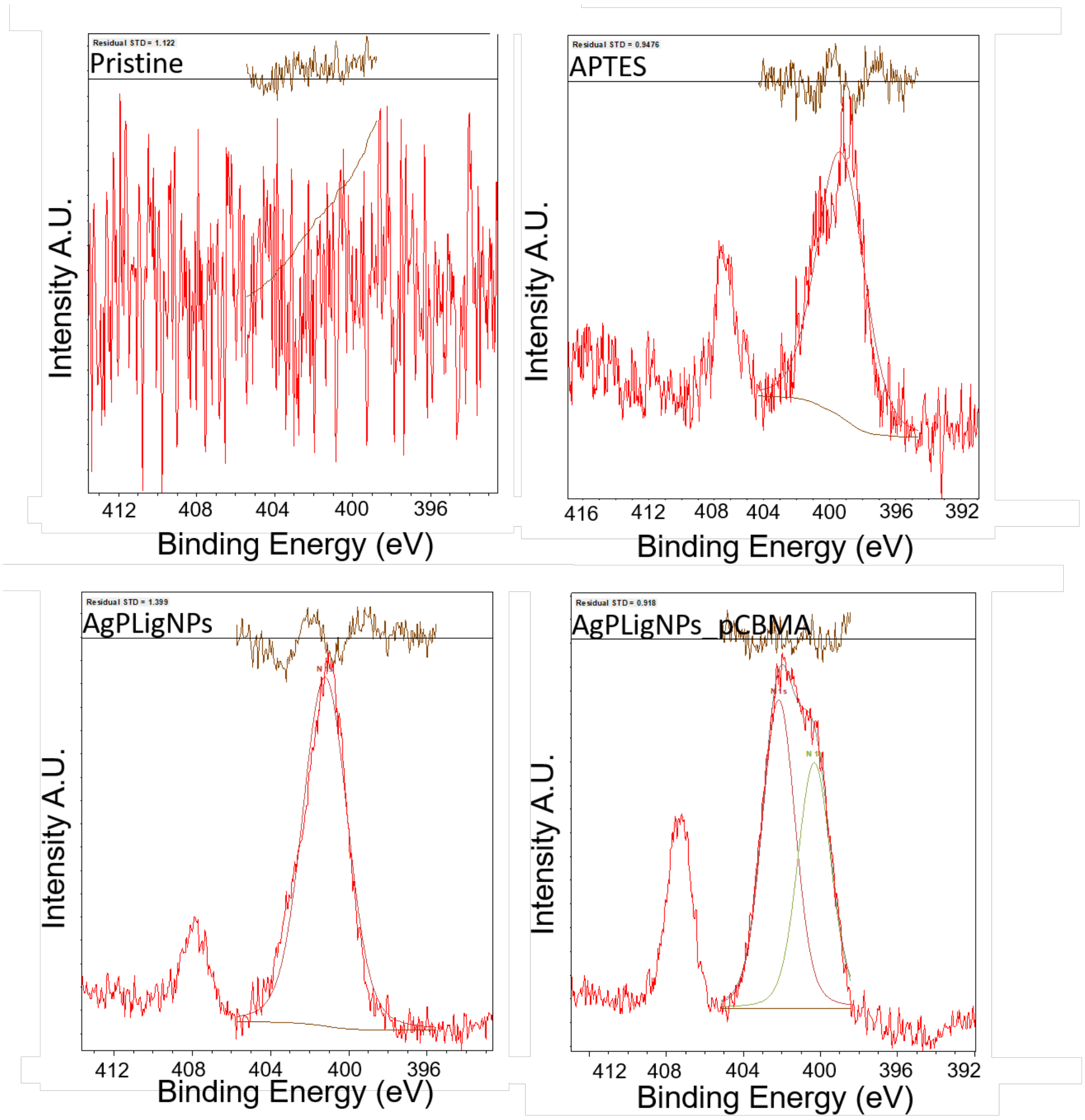

Figure S5. High-resolution N1s XPS spectra of pristine silicone, pre-aminated silicone (APTES), AgPLigNPs-coated and AgPLigNPs_pCBMA-coated silicone.

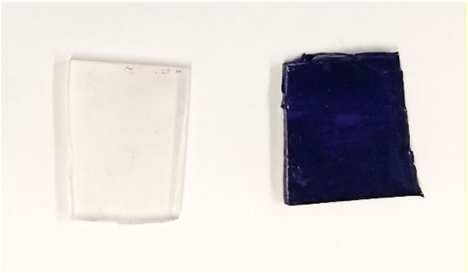

Figure S6. Pristine and aminated PDMS after ninhydrin assay.

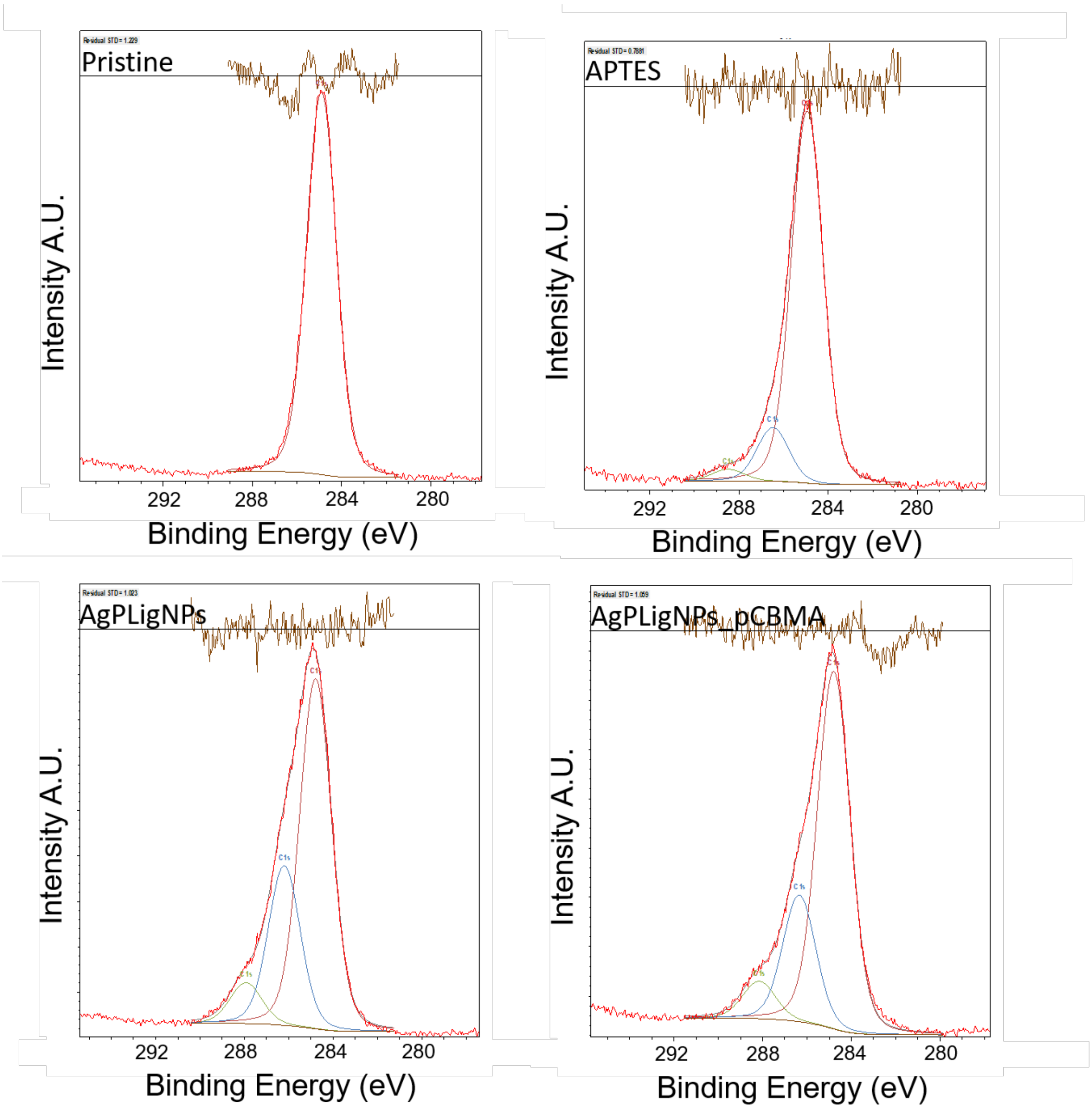

Figure S7. High-resolution N1s XPS spectra of pristine silicone, pre-aminated silicone (APTES), AgPLigNPs-coated and AgPLigNPs_pCBMA-coated silicone.

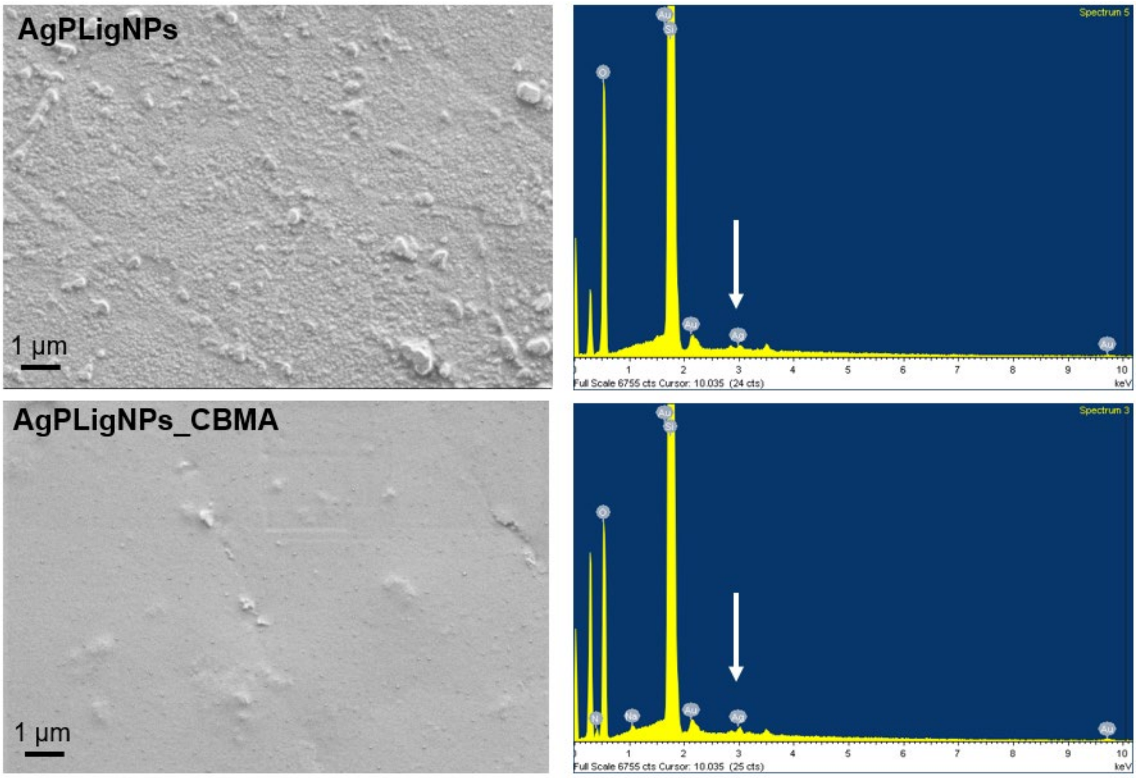

Figure S8. SEM images and EDX data of AgPLigNPs and AgPLigNPs_CBMA coatings.

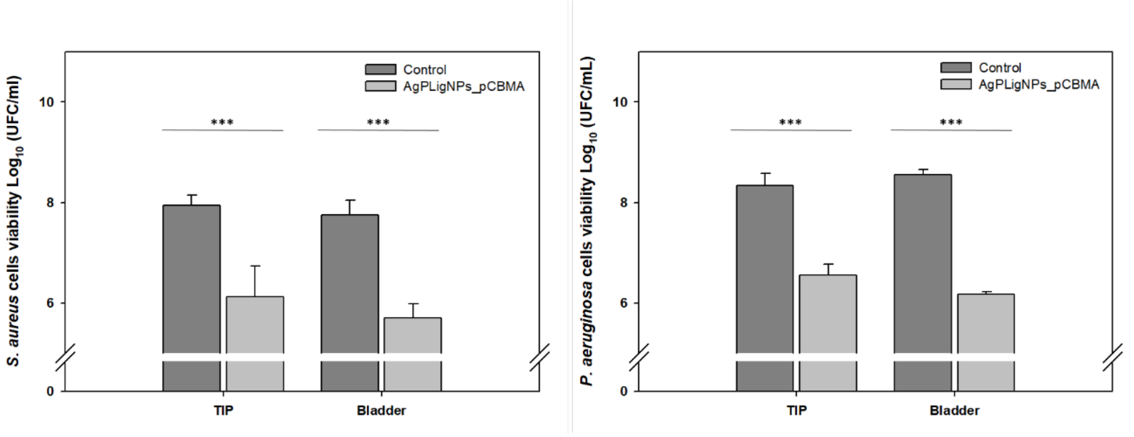

Figure S9. Hydrodynamic antibiofilm test using individual cultures.

Table S1. N1s deconvoluted XPS spectra and functional group assignment.

N 1s	Atomic composition (%)	
Peak position (eV)	**400**	**402.2**
	**N-H/N-R**	**N^{+}-R_4**
**Pristine**	0	0
**APTES**	100	0
**AgPLigNPs**	100	0
**AgPLigNPs_pCBMA**	44.35	55.65

Table S2. C1s deconvoluted XPS spectra and functional group assignment.

C 1s	Atomic composition (%)		
Peak position (eV)	**284.8**	**288.5**	**288**
	**C-C/C-H**	**C-O-C/C-OH**	**COOH/COOR**
**Pristine**	100	0	0
**APTES**	85.08	2.66	12.26
**AgPLigNPs**	68.58	24.23	7.19
**AgPLigNPs_pCBMA**	63.61	28.98	7.41

Table S3. Blood parameters (rabbits 1–3 uncoated and rabbits 4–6 coated catheters) on the first day of catheterisation. White blood cell count (WBC), Lymphocyte count (Lymph.), Monocyte count (Mon.), Granulocyte count (Gran.), Red blood cell count (RBC), Haemoglobin (HGB), Platelet count (PLT).

**Parameters**	**Ref. Range**	**Rabbit 1**	**Rabbit 2**	**Rabbit 3**	**Rabbit 4**	**Rabbit 5**	**Rabbit 6**
WBC 10^9/L	5.2–13.5	10.7	**H 17.8**	12.8	10.0	11.5	12.5
Lymph. 10^9/L	3.2–9.0	3.9	**H 9.5**	5.3	3.4	3.5	4.5
Mon. 10^9/L	0.1–0.6	0.6	0.3	0.5	0.6	0.5	0.5
Gran. 10^9/L	2.0–7.5	6.2	**H 8.0**	7.0	6.8	6.6	6.3
RBC 10^{12}/L	5.00–7.60	5.8	6.9	7.3	6.8	6.6	6.3
HGB g/L	320–370	340	350	360	330	320	320
PLT 10^9/L	250–650	380	420	400	400	370	350

Table S4. Blood parameters on 7th day of catheterisation, corresponding to Table S3.

Parameters	Ref. Range	Rabbit 1	Rabbit 2	Rabbit 3	Rabbit 4	Rabbit 5	Rabbit 6
WBC 10^9/L	5.2–13.5	8.4	13.4	10	8.5	9.0	8.5
Lymph. 10^9/L	3.2–9.0	**L 2.9**	5.5	**L 3.0**	**L 3.1**	3.0	**L 3.0**
Mon. 10^9/L	0.1–0.6	0.3	0.5	0.5	0.3	0.5	0.5
Gran. 10^9/L	2.0–7.5	5.2	7.4	6.5	5.1	5.2	5.0
RBC 10^{12}/L	5.00–7.60	**L 4.9**	5.0	**L 4.7**	**L 4.9**	5.2	**L 4.8**
HGB g/L	320–370	**L 113**	**L 200**	**L 111**	**L 118**	**L 236**	**L 215**
PLT 10^9/L	250–650	**H 1308**	**H 925**	**H 778**	**H 686**	**H 678**	**H 732**

Table S5. Blood biochemical parameters (rabbits 1–3 uncoated and rabbits 4–6 coated catheters). S_Albumin (ALB), Total Protein (TP), Globulin (GLO), Calcium (Ca), Glucose (Glu), Blood urea nitrogen (BUN), Phosphorus (P), Amylase (AMY), Cholesterol (CHOL), Alanine transaminase (ALT), Total bilirubin (TBIL), Alkaline phosphatase (ALP), Creatinine (CRE), Creatine kinase (CK).

Parameters	Ref. Range	Rabbit 1	Rabbit 2	Rabbit 3	Rabbit 4	Rabbit 5	Rabbit 6
ALB	27–50 g/L	42.9	33.9	42.0	42.4	40.6	45.4
TP	49–71 g/L	62.0	60.0	62.1	60.9	55.8	65.9
GLO	15–33 g/L	19.1	26.1	20.1	18.5	15.2	20.5
Ca	2.2–3.9 mmol/L	3.5	3.4	3.0	3.45	3.5	3.0
GLU	5.5–8.2 mmol/L	**H 13.82**	**H 14.0**	**H 13.0**	**H 14.59**	**H12.2**	**H 13.0**
BUN	10.1–17.1 mmol/L	**L 7.02**	**L 8.0**	**L 7.06**	**L 7.01**	**L 8.03**	**L 7.8**
P	1–2.2 mmol/L	1.19	1.2	1.1	1.25	1.2	1.4

AMY	212–424 U/L	**L 138**	**L 135**	**L 188**	**L 135**	**L 170**	**L 165**
CHOL	0.1–2 mmol/L	1.11	1.13	0.9	1.13	1.0	0.9
ALT	27.4–72.2 U/L	56	66	63	53	45	48
TBIL	2.6–17.1 µmol/L	2.97	3.7	2.8	2.92	5.0	3.7
ALP	12–96 U/L	51	61	72	60	54	60
CRE	74–1711µmol/L	**L 47**	**L 59**	**L 64**	**L 60**	**L 67**	**L 70**
CK	58.6–175 U/L	**H 1739**	**H 1772**	**H 1800**	**H 1770**	**H 1800**	**H 1860**

Table S6. Urinalysis (rabbits 1–3 uncoated and rabbits 4–6 coated catheters) on 7th day of catheterisation. White blood cells (WBC), Ketone levels (KET), Nitrites (NIT), Urobilinogen (URO), Bilirubin (BIL), Glucose (GLU), Protein levels (PRO), Urine specific gravity (SG), pH, Blood cells (BLD), urine vitamin C (Vc), Microalbuminuria (Ma), Calcium (Ca), Creatinine (CR).

**Parameters**	**Rabbit 1**	**Rabbit 2**	**Rabbit 3**	**Rabbit 4**	**Rabbit 5**	**Rabbit 6**
WBC cell/µL	+1 70	15	15	15	0	0
KET mmol/L	± 0.5	± 0.5	± 0.5	0	0	0
NIT	-	-	-	-	-	-
URO umol/L	+1 33	-	+1 33	-	-	-
BIL µmol/L	+1 8.6	0	+1 8.6	0	0	0
GLU mmol/L	0	0	0	0	0	0
PRO g/L	+3 ≥3.0	+1 0.3	+1 0.3	+1 0.3	0	0
SG	1.005	1.005	1.010	1.005	1.010	1.005
pH	8.0	8.0	8.0	8.0	8.0	8.0
BLD cell/µL	0	0	0	0	0	0
Vc mmol/L	0	0	0	0	0	0
MA mg/L	≥150	≥150	≥150	≥150	≤10	≤10
Ca mmol/L	7.5	7.5	7.5	7.5	7.5	7.5
CR mmol/L	≥26.4	≥26.4	≥26.4	17.6	≥26.4	≥26.4
